# Dual targeting of oncogenic microtubules and mitochondria in PDAC

**DOI:** 10.18632/oncoscience.641

**Published:** 2026-01-28

**Authors:** Michael W. Spinrad, Chun Cai, Lauren C. Gattie, Rui Wang, Aman Bajwa, Wei Li, Evan S. Glazer

**Affiliations:** ^1^Department of Surgery, The University of Tennessee Health Science Center, Memphis, TN 38163, USA; ^2^Center for Cancer Research, College of Medicine, The University of Tennessee Health Science Center, Memphis, TN 38163, USA; ^3^Department of Pharmaceutical Sciences, College of Pharmacy, The University of Tennessee Health Science Center, Memphis, TN 38163, USA

**Keywords:** pancreatic ductal adenocarcinoma, bromo- and extra-terminal domain, mitochondrial stress, mitophagy, mitochondria

## Abstract

Pancreatic ductal adenocarcinoma (PDAC) is one of the deadliest cancers. Microtubule inhibition is a promising therapeutic target as microtubule dynamics play a critical role in growth of metastases due to over expression of several β-tubulin subtypes compared to normal cells. Previous studies have shown that decreased expression of βIII- and βIVb-tubulin is associated with decreased PDAC cell growth. Bromodomain and Extra-Terminal domain (BET) proteins are transcription factors that regulate mitochondria proteins. In this study, we hypothesize that SB-216 and Veru-111 (related novel compounds) inhibit cell growth via suppression of oncogenic βIII- and βIVb-tubulin subtypes and mitochondria function via suppression of BRD4, the most active BET protein. PDAC cell growth was analyzed with the IncuCyte Live-Cell Analysis system. mRNA expression of βIII- and βIVb-tubulin was evaluated with quantitative real time PCR. Western blot analysis was performed for βIII, βIVb-tubulin, and BRD4 protein expression and expression of autophagy and mitophagy markers LC3B and p62/SQSTM1. Mitochondrial function/respiration was measured using a Seahorse XF-24 Flux Analyzer. Cell growth was greatly inhibited across all doses in multiple PDAC cell lines (*p* < .0001). mRNA expression of *TUBB3* (βIII subtype) and *TUBB4* (βIVb subtype) was significantly decreased (*p* < .05). BRD4 protein expression was reduced in with compensatory increase in mRNA expression. Treated PDCL had reduced mitochondrial respiration. Autophagy markers were increased in treated PDAC cells. Our data demonstrates that SB-216 effectively inhibits PDAC cell growth through inhibiting oncogenic microtubules and mitochondrial function. This novel approach simultaneously targets two hallmarks of cancer and patient demise.

## INTRODUCTION

Pancreatic ductal adenocarcinoma (PDAC) is the fourth leading cause of cancer related deaths worldwide, with incidence rates increasing annually [1]. Treatment options are limited due to high chemotherapy resistance and patients often present with metastatic disease, leading to overall very poor prognosis and patient death [2].

Microtubule targeting agents (MTA) have been a mainstay treatment for cancers since the approval of the taxane Paclitaxel in 1984 [3]. Paclitaxel is a key component in one of the standards of care treatments for patients with PDAC (in combinations with gemcitabine) [4]. Unfortunately, many cancers develop resistance to Paclitaxel, including PDAC [5]. The mechanism of taxane resistance is thought to be the result of overexpression of specific β-tubulin isotypes, notably βIII-tubulin (TUBB3) and βIVb-tubulin (TUBB4), which is upregulated in PDAC compared to normal pancreas cells and thus are potential therapeutic targets [6–8].

Compared to targeting microtubules, disruption of cellular metabolism as an anti-cancer treatment strategy is relatively novel [9–11]. Although not well studied, there is recent evidence showing that mitochondrial dynamics (metabolism) play a key role in the development of PDAC [12]. Mitochondrial function is involved in cellular growth, inflammation, and the development of the tumor microenvironment in PDAC [13]. Specifically targeting mitochondrial metabolism, however, is difficult due to significant tumor heterogeneity [14]. In addition, there are links between microtubules and mitochondria in normal cellular function and programmed cell death mechanisms suggesting an ideal therapeutic target [15, 16]. Our group was the first to identify that inhibition of bromo-domain related protein 4 (BRD4), a member of the bromo- and extra-terminal domain (BET) protein family led to decreased mitochondrial function and cell death in PDAC models [11].

While not directly targeting mitochondria, MTAs induce cell cycle arrest and have been shown to trigger apoptosis through caspase activation via the intrinsic apoptotic pathway, which is closely linked to mitochondrial membrane permeabilization [15]. Currently, there is ongoing research to develop new colchicine binding microtubule inhibitors, which have been shown to be less prone to chemoresistance than currently available taxanes and vinca alkaloids [17]. Recently, our group developed novel colchicine binding microtubule inhibitors, SB-216 and Veru-111, which have enhanced binding characteristics to oncogenic microtubule subtypes and induce mitochondrial mediated apoptosis [18]. Our group improved on Veru-111 to develop enhanced drug-like properties and created SB-216 [19]. We hypothesize that SB-216 has improved anti-microtubule and anti-mitochondria function in PDAC models compared to VERU-111.

## RESULTS

### SB-216 reduces cell growth

**Figure 1:**
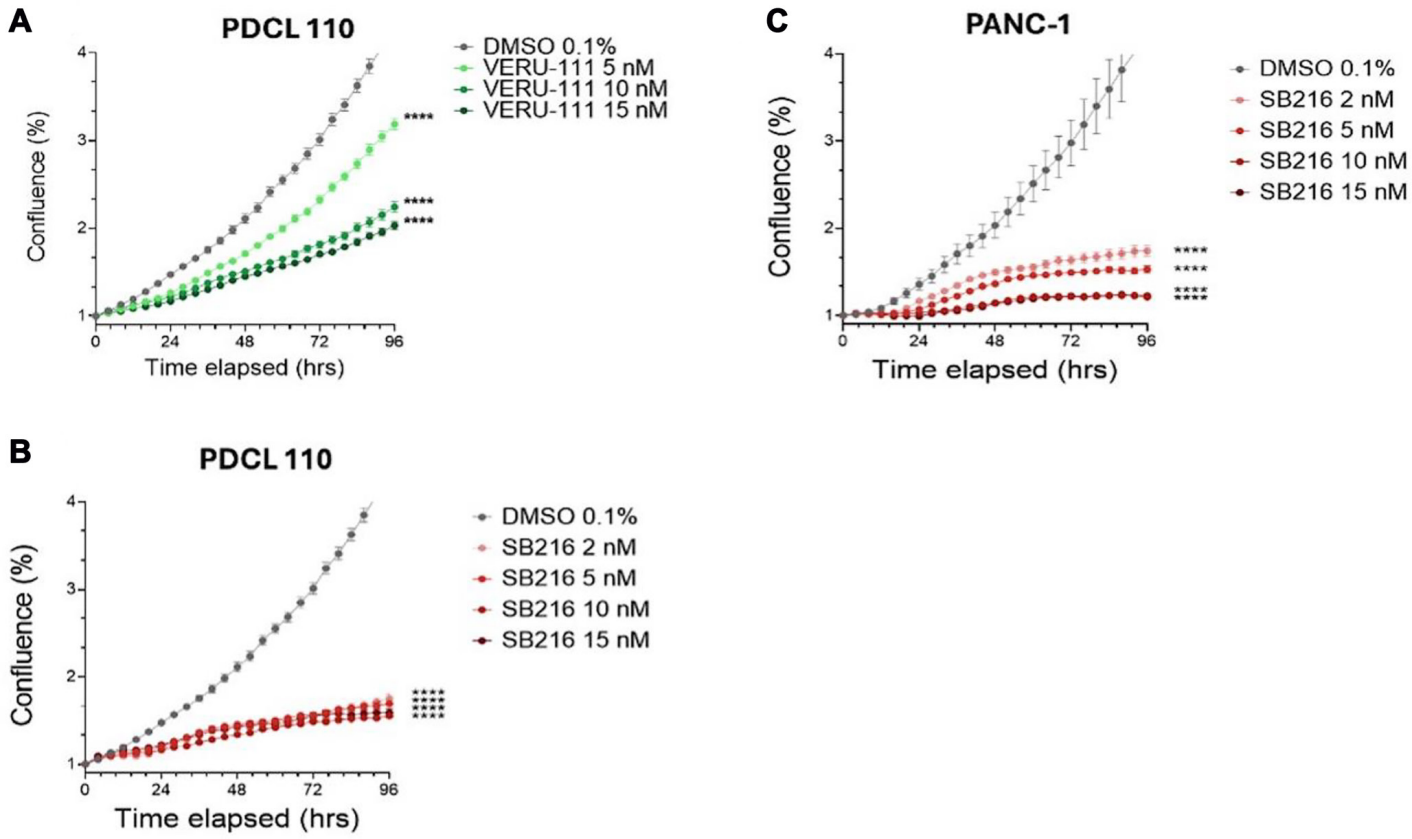
SB-216 significantly inhibits growth of pancreatic ductal adenocarcinoma cells. VERU-111 inhibits growth of (**A**) Patient-derived PDAC cell line (PDCL) 110 in a dose- dependent manner. SB-216 is more effective than Veru-111 at inhibiting growth of PDCL 110 cells (**B**) and Panc-1 cells (**C**). Cells were seeded (6000 cells/well) and treatment was administered after 24 hours. Plates were analyzed overtime with phase contrast images of confluency (Value means ± SEM, *n* = 8), Asterisks represent a significant reduction in cell growth (^**^p < 0.01, ^****^p < 0.0001). The Incucyte Live-Cell Imaging Analysis System was utilized.

We first evaluated the effects of SB-216 on the cell growth of Panc-1 and patient-derived human PDAC cell lines (PDCLs) by exposing cells to increasing concentrations of SB-216 (2–15 nM). SB-216 inhibited cell growth in a dose dependent manner, with 15 nM having the largest decrease in growth and 2 nM having the lowest decrease compared to the control (Figure 1A–1C). This was confirmed in different cell lines. Panc -1 (Figure 1A, *p* < 0.0001 for all doses) and PDCL 110 (Figure 1B, *p* < 0.0001 for all doses). Similarly, VERU-111 (5 nM–15 nM) inhibits cell growth in PDCL 110 cells in a dose dependent manner (Figure 1C, 10–15 nM *p* < 0.0001). Compared to the 15 nM dose of VERU-111, SB-216 demonstrated a greater decrease in cell growth (15 nM, *p* = 0.004).

### Inhibition of BRD4 protein expression and decrease cellular respiration

**Figure 2:**
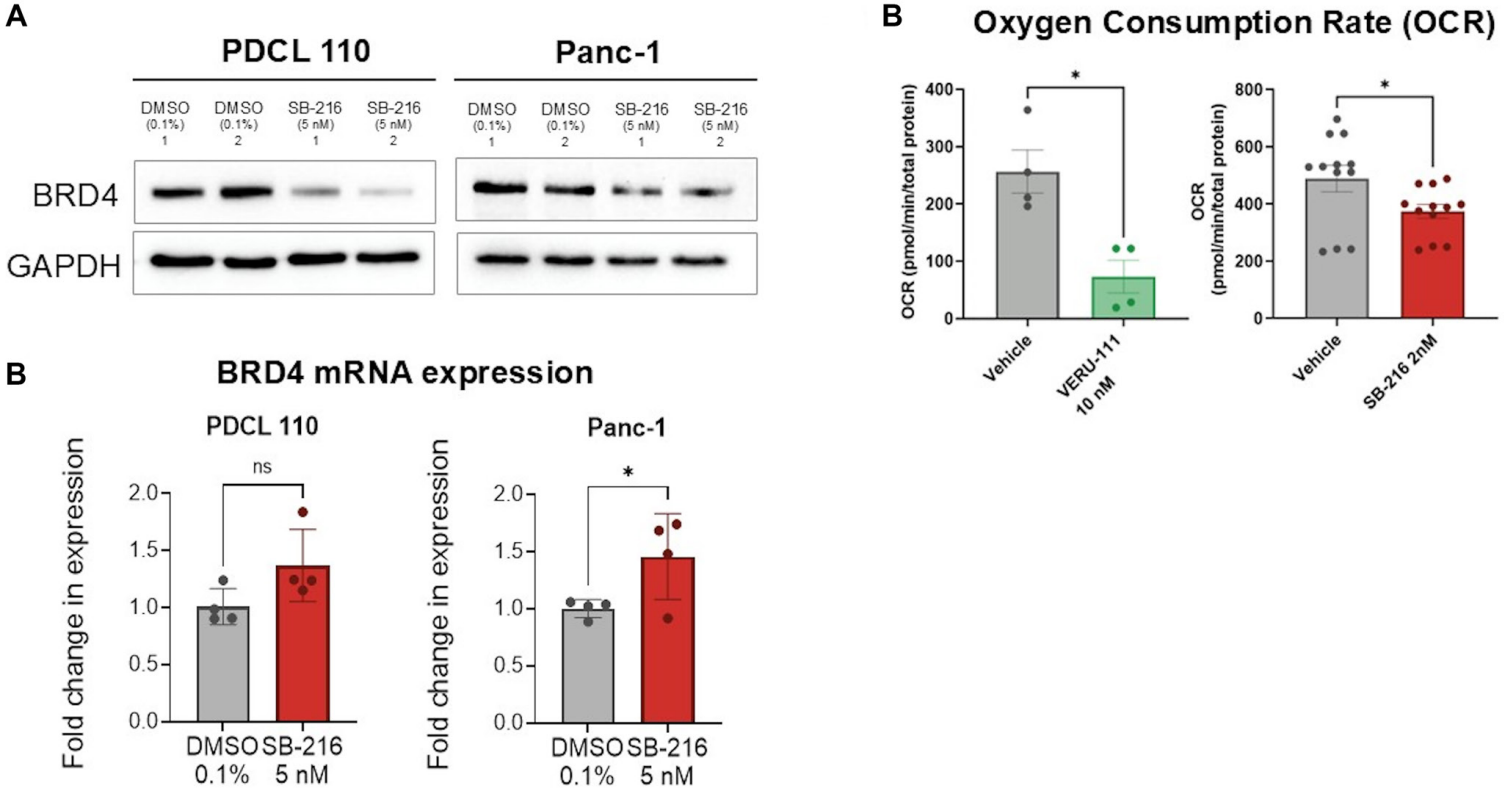
SB-216 significantly reduced the protein expression of BRD 4 and disrupts oxygen consumption in pancreatic cancer cells. (**A**) SB-216 decreased protein expression of BRD4 in both PDCL110 and Panc-1 PDAC cells. (**B**) SB-216 increased mRNA expression of *BRD4* in PDCL 110 and Panc-1 cells. (**C**) Both Veru-111 (10 nM, 72 hours) and SB-216 (2 nM, 24 hours) significantly decreased the oxygen consumption rate (OCR) of PDCL 110 PDAC cells compared to DMSO vehicle control. Mitochondrial OCR was performed using a Seahorse XFe24 analyzer measuring different bioenergetic parameters of oxygen consumption rate (OCR) (Values are mean ± SEM, *n* ≥ 4,12 ^*^p < .05). Protein expression was evaluated with western blot. GAPDH was used as the loading control. qPCR was performed to determine mRNA expression. Actin was used as an internal control. Graphs represent relative fold change of expression mRNA. (Values mean ± SEM, *n* = 4, ^*^p < 0.05).

We evaluated the role of SB-216 in mRNA and protein expression of BRD4. Western blot analysis shows a decrease in BRD4 protein expression in both PDCL-110 and Panc-1 cell lines upon treatment (Figure 2A). With qPCR, we then found a statistically significant increase in *BRD4* mRNA expression upon experimental treatment of Panc-1 cells (Average increase in fold expression 0.4542 ± 0.192 *p* = 0.028, Figure 2B). We further investigated disruption of mitochondrial function with VERU-111 and SB-216. Mitochondrial oxygen consumption rate (OCR) was assessed using a Seahorse XF Analyzer. A mitochondrial stress test was performed on PDCL-110 cells treated with VERU-111 (10 nM) for 72 hours and SB-216 (2 nM) for 24 hours (Figure 2C) based on time to effect previously seen (data not shown). Cells treated with VERU-111 consumed an average 183.7 pmol oxygen/min less than the control (*p* = 0.03). Cells treated with SB-216 consumed an average 114.8 pmol oxygen/minute less than the control (*p* = 0.04).

### SB-216 inhibits mRNA and protein expression of oncogenic β-tubulin subtypes

**Figure 3:**
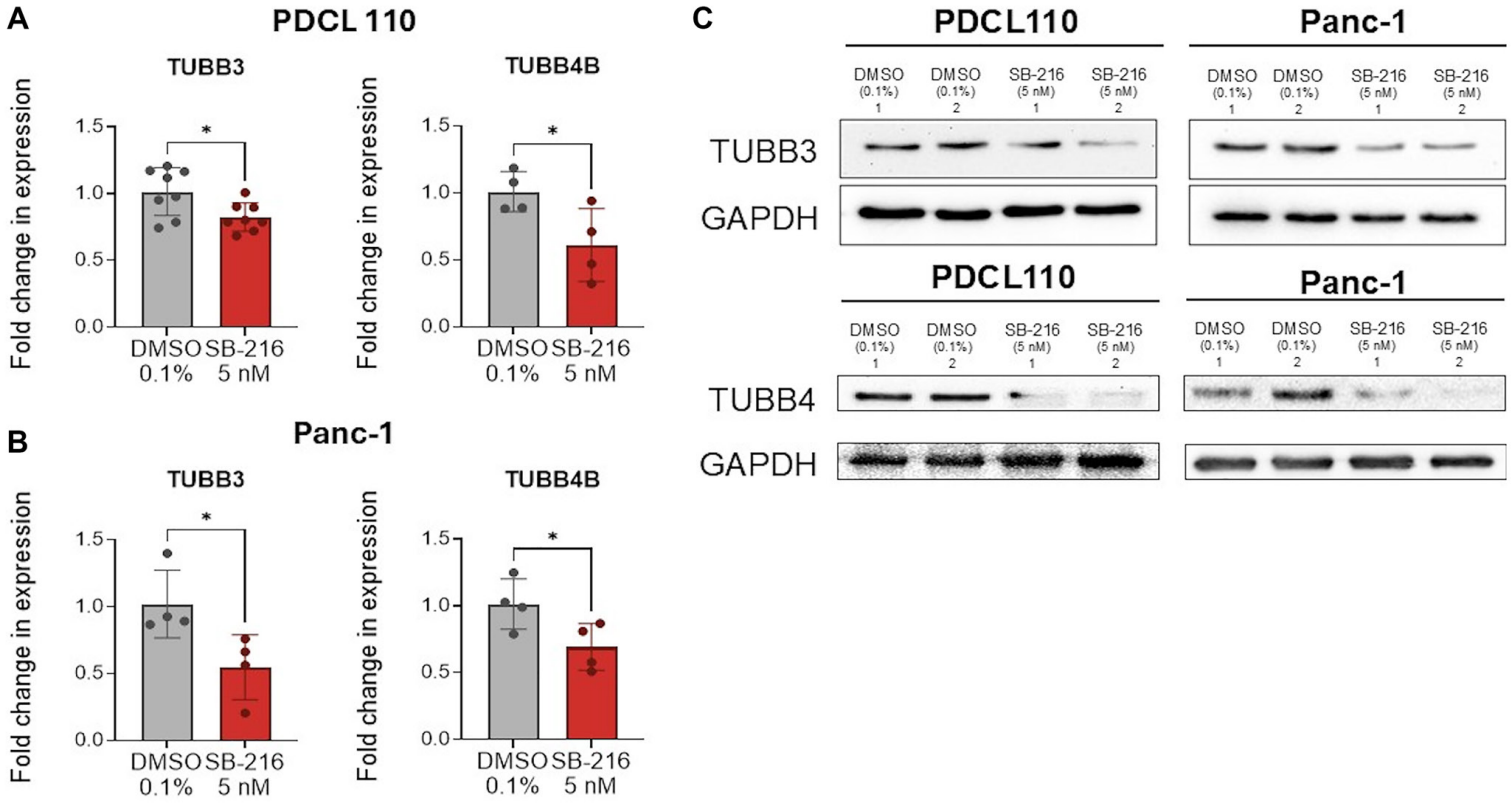
SB-216 significantly reduces mRNA expression of oncogenic tubulin subtypes βIII and βIVB and protein expression of βIII. SB-216 reduces mRNA expression of TUBB3 and TUBB4B in (**A**) PDCL110 and (**B**) Panc-1 cells. (**C**) SB-216 reduces protein expression of TUBB3 and TUBB4B. PDCL110 and Panc-1 cells were treated with SB-216 (5 nM) or DMSO (control) for 48 hours. RNA was isolated and transcribed for cDNA preparation. qPCR was performed to determine mRNA expression. Actin was used as an internal control. Graphs represent relative fold change expression of each tubulin mRNA expression. (Values mean ± SEM, *n* = 8,4). PDCL110 and Panc-1 cells were treated with SB-216 (5 nM) or DMSO (control) for 48 hours. Protein expression was evaluated with western blot. GAPDH was used as the loading control (and is same from Figure 2A). Asterisks represent a significant reduction in mRNA expression (^*^p < 0.05). Panc1 TUBB3 protein blot was the same as BRD4 blot with the same loading control GAPDH as on Figure 2A.

We next evaluated the effects of SB-216 as a tubulin inhibitor in PDAC cell lines. qPCR analysis shows a statistically significant reduction in mRNA expression of oncogenic β-tubulin subtypes βIII, in both PDCL-110 (Figure 3A, average fold reduction of 0.19 ± 0.07, *p* = .02) and Panc-1 (Figure 3B, average fold reduction of 0.47 ± 0.17, *p* = .03) cells. There was a similar reduction in mRNA expression of tubulin β-IVb in PDCL 110 (Figure 3A, average fold reduction 0.39 ± 0.15, *p* = 0.04) and Panc -1 cells (Figure 3B, average fold reduction of 0.32 ± 0.13, *p* = 0.04). Western blot analysis further validated this with protein inhibition of tubulin βIII and tubulin βIVB in the Panc-1 cell line (Figure 3C).

### SB-216 increases autophagy markers in PDAC cells

**Figure 4:**
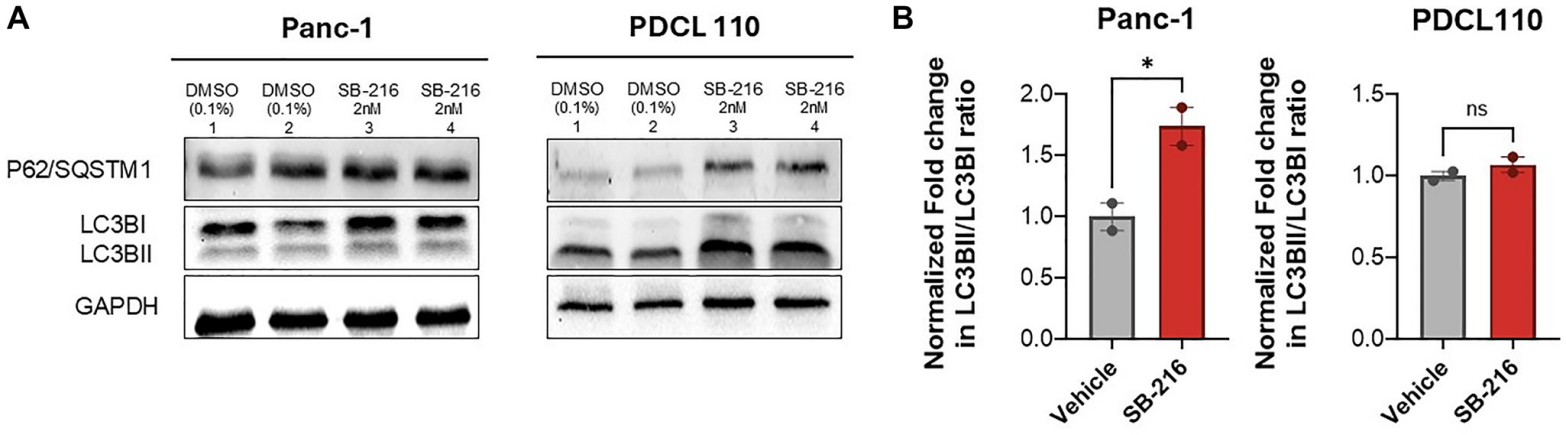
SB-216 induces autophagy in pancreatic cancer cells. (**A**) Protein expression was evaluated with western blot (*n* = 2), GAPDH was used as the loading control. Panc-1 and PDCL 110 cells were treated for 48 hours with SB-216 (2 nM) or DMSO (Control). (**B**) A quantitative assessment of protein intensity was performed using ImageJ software. Graphs represent fold change in LC3BII band intensity relative to LC3BI, normalized to GAPDH band intensity. (Values mean ± SEM, *n* = 2, ^*^p < 0.05).

To further investigate the effects of SB-216 on cellular metabolism and mitochondrial injury, we investigated the expression of LC3BI, LC3BII, and p62/SQSTM1. Western blot analysis revealed an increase in LC3BI and LC3BII protein expression in both Panc-1 and PDCL-110 cell lines upon treatment with 2 nM SB-216 compared to DMSO control treatment (Figure 4A). The ratio of LC3BII to LC3BI had a 0.74 ±.1 (*P* = 0.03) fold increase in Panc-1 cells treated with SB-216 (Figure 4B). Additionally, PDCL-110 cells treated with SB-216 demonstrate a relatively higher accumulation of p62/SQSTM1 than seen in Panc-1 cells.

## DISCUSSION

PDAC is a deadly disease with poor outcomes and limited treatment options [1]. Current treatment options, such as gemcitabine-nab-paclitaxel for PDAC are limited by high chemoresistance and poor tumor penetration [20] while treatments such as FOLFIRINOX are quite toxic [21]. Recent research has shown colchicine binding site microtubule inhibitors to be a promising class of therapeutic agents with anti-cancer properties, often as dual- target inhibitors leading to single-agent combinational strategies [17, 22]. In our current study, we demonstrate two related compounds control PDAC cells by targeting microtubule and mitochondrial regulatory framework. We have shown that SB-216, a derivative of Veru-111, has increased and prolonged inhibition of cell growth in PDAC cells through down regulation of oncogenic β-tubulin and BRD4 protein expression with subsequent decreased metabolic activity (oxygen consumption).

BRD4 (and all BET proteins) has several functions within the cell as a transcription factor [23]. BET inhibitors are a new class of anti-cancer therapeutics currently in development with multiple ongoing clinical trials [24]. Specifically, prior research shows that BET inhibitors reduce cell viability, disrupt the cell cycle, and cause significant mitochondrial dysfunction in multiple PDAC cell lines [11]. In response to loss of BRD4 protein with SB-216 treatment, PDAC cells attempt to compensate by increasing gene expression of BRD4 but cannot sufficiently respond based on maintained inhibition of cell growth and loss of mitochondrial respiration. In addition, different cell lines (e.g., Panc-1 and PDCL-110) also respond differently. We theorize that this is based on difference in metabolism (mitochondrial regulation) in each cell line. While specific protein response may be slightly different, the overall change in metabolic/mitochondrial programming remains consistent.

Advanced and aggressive tumors, such as PDAC, not only regulate their own TME but also affect whole host (patient) homeostasis to support cancer growth [25]. This includes mitochondria in muscle inducing cachexia and a variety of hormonal changes inducing a wide variety of cancer treatment-related clinical syndromes [26–28]. These homeostatic imbalances are due to cancer alone, chemotherapy treatment alone, and the combination of the two [29, 30]. We theorize that reprogramming of mitochondrial will help normalize host bioenergetics although we have data suggesting that compensatory effects (such as mRNA expression of *BRD4*) will play an important role which we explore in future analyses.

As previously reported, VERU-111 reduces cell viability and preferentially binds to oncogenic tubulin subtypes βIII and βIVb [18]. Here, we demonstrate that SB-216 significantly inhibits cell growth in PDAC cells, both in commercial and PDCL, demonstrating the potent utility of SB-216. We then showed that that SB-216, like VERU-111, significantly reduces mRNA expression of *TUBB3* and *TUBB4*, as well as reduces the protein expression of TUBB3. This has important implications as a new therapeutic target. TUBB3 and TUBB4 are highly upregulated in PDAC cells compared to normal pancreatic cells and are implicated in the development of paclitaxel resistance [7, 31]. By selectively binding oncogenic tubulin subtypes, SB-216 may preferentially target PDAC cells, increasing effectiveness and reducing systemic toxicity.

We hypothesized that our novel class of microtubule inhibitor would also inhibit mitochondrial function by acting as a dual-target inhibitor. Here we show that VERU-111 and SB-216 significantly reduce oxygen consumption rate in PDAC cells favoring mitophagy, specifically, as the mechanism of action over autophagy. We also show inhibition of BRD4 protein and expression with a compensatory increase in mRNA expression seen with treatment at a single time point. Dual targeting of BRD4 or sequential dosing may drastically increase the potency of SB-216 as an anti-cancer drug. Given the increase in mRNA expression as a compensatory mechanism, we also theorize that this will become a relevant resistance mechanism clinically. By simultaneously targeting microtubules and inducing mitophagy, this treatment strategy will possibly avert this predictable resistance mechanism that has been suggested [32, 33].

We also investigated the effect of SB-216 on autophagy in general. Briefly, autophagy is a highly conserved cellular process involved with cellular maintenance and degradation [34]. The role of autophagy in PDAC has not been fully elucidated but multiple mechanism altering autophagosome biology lead to cellular death. Evidence suggests that PDAC cells have elevated autophagy which are associated with worse prognosis [35]. However, autophagy is context dependent and can act as a tumor suppressor or promote tumor survival whereas mitophagy is generally detrimental to the tumor. Some studies suggest that gemcitabine induces autophagy and suppresses cancer [36, 37]. Other studies report that chemotherapy induced autophagy may enhance tumor survival in PDAC [38]. In the current study, we show that SB-216 treated Panc-1 cells accumulate LC3BII and P62, both markers for autophagy (Figure 4). LC3BII is associated with autophagosome formation, whereas P62 is associated with LC3BII and marks cell products for degradation [39, 40]. An accumulation of LC3BII indicated an increase in autophagosomes [41]. Under normal conditions, P62 is degraded as part of the pathway [42]. In the presence of SB-216, it is possible that the later phases of autophagy are inhibited, preventing the localization of P62 to the autophagosome. Alternatively, longer exposure to SB-216 may be needed for autophagy induction, but this overall leads to cell death, and since it alters oxygen metabolism, this supports that ultimately these drugs leads to mitophagy-mediated cell death.

Our work has limitations. First, experiments were carried out *in vitro* on cell cultures. Future studies will utilize *in vivo* models as well. The model systems and assays utilized (i.e., SeaHorse instrument) have artificial, experimental limitations that prevent complete mechanistic understanding. The Mito Stress Test used to measure oxygen consumption is limited to *in vitro* experiments and is reliant on cell membrane permeability for drug delivery, which may affect results on different cell lines. Second, more research is required to investigate the relationship between SB-216 and autophagy especially using *in vivo* models. The present study has demonstrated that SB-216 may have an effect on autophagy in PDAC cells, however the results are limited because autophagy flux is not measured and mitophagy may be the dominant mechanism of action. Future studies will explore the impact SB-216 has on autophagy with flux assays, as well as fluorescent microscopy to directly visualize mitochondria and autophagosomes in context of mitochondria injury. Demonstrating combined microtubule and metabolism inhibition strengthens the potential of SB-216 as a therapeutic agent, however the present study is unable to elucidate the mechanism of how SB-216 affects mitochondrial function outside of BRD4, a regulatory transcription factor of mitochondrial complex transcription. Lastly, while dual-targeting microtubule function and mitochondrial function may produce powerful anti-cancer effects, there is also potential for severe toxicity that will need to be accounted for with *in vivo* models and early phase clinical trials. Based on our initial experience with these drugs, we are biased that SB-216 will lead to a better cancer therapeutic but this is not conclusive.

## MATERIALS AND METHODS

### Preparation and collection of PDAC cell lines

PANC-1 (ATCC, Manassas, VA, USA), and patient derived cell line PDCL 110 (69 year-old African-American female, PDAC stage ypT2N0M0 after 4 cycles of FOLFIRONOX neoadjuvant chemotherapy) were grown in DMEM/F12 (HyClone, UT, USA) medium supplemented with 10% exosome-depleted FBS (SystemBio, CA, USA), 100 U/ml penicillin, and 100 g/ml streptomycin at 37°C with 5% CO_2_. The patient-derived PDAC was obtained from a surgical specimen by one of the authors (ESG) with ethical approval from The University of Tennessee Health Science Center Institutional Review Board approved (protocol #17-05064-XP) following the Declaration of Helsinki and other ethical guidelines. Patients provided signed informed consent for their specimens to be involved in this research. Specimens were prepared for storage and culture immediately after acquisition during surgery. These were used during passage 1 or 2 after collection. ATCC cell lines were obtained directly from ATCC for use and all cell lines were tested annually for mycoplasma contamination (PCR method) and authenticated by STR profiling, SB-216 and VERU-111 were created in Dr. Li’s laboratory per published protocol [19, 43]. Doses of SB-216 and VERU-111 used were chosen based on previous experience and preliminary evaluation (data not shown).

### Cell growth assays

The growth and proliferation of PDAC cell lines were measured in real time at 4-hour intervals in replicates of 8 wells/group using high resolution bright-field imaging of live cells on an IncuCyte instrument (Sartorius, Ann Arbor, MI, USA). IncuCyte software was used to analyze and quantify results. The IncuCyte analysis measures cell growth, proliferation, and morphology.

### Quantitative reverse transcription polymerase chain reaction (qPCR)

Total RNA was isolated using Trizol reagent following the manufacturer’s protocol. cDNA was synthesized from 1 μg of total RNA using an iScript cDNA Synthesis Kit, and qPCR was performed using SsoAdvanced Universal SYBR Green Supermix in a CFX96 Touch Real-Time PCR Detection System (Bio-Rad). Relative gene expression was normalized to β-actin as the internal control, and fold changes were calculated using the 2^−ΔΔCt^ method. The following primer sequences were used:

BRD4 forward: 5′-CGCTATGTCACCTCCTGTTTGC-3′
BRD4 reverse: 5′-ACTCTGAGGACGAGAAGCCCTT-3′
TUBB3 forward: 5′-GGCCAAGGGTCACTACACG -3′
TUBB3 reverse: 5′-GCAGTCGCAGTTTTCACACTC-3′
TUBB4 forward: 5′-GGACAACTTCGTTTTCGGTCA-3′
TUBB4 reverse: 5′-CCTTTCTCACAACATCCAGCAC-3′
β-actin forward: 5′-TGACGTGGACATCCGCAAAG-3′
β-actin reverse: 5′-CTGGAAGGTGGACAGCGAGG-3′.

### Western blot protein analysis

Cultured cells were lysed in radioimmunoprecipitation assay (RIPA) buffer (Thermo Fisher Scientific, Waltham, MA, USA). The resulting whole-cell lysates were supplemented with 1 mM phenylmethylsulfonyl fluoride (PMSF; Cell Signaling Technology) protease inhibitor and phosphatase inhibitor cocktail (Sigma-Aldrich), according to the manufacturer’s protocol. The total protein concentration was determined using a standard Bradford assay (Bio-Rad). For each sample, 25 μg of total protein was separated on a 10% (BRD4, TUBB3, TUBB4) or 15% (p62, LC3B-II) PAGE gel and transferred to polyvinylidene difluoride (PVDF) membrane (Bio-Rad) with wet transfer (BRD4) or with the Trans-Blot turbo Transfer System (Bio-Rad) (TUBB3, TUBB4, p62, LC3B-II). The membranes were blocked in TBST buffer with 5% non-fat milk for 1 h and incubated at 4 °C overnight with the appropriate primary antibodies: LC3B-II (1:1000 dilution), p62 (1:2000), BRD4 (1:2000), TUBB3(1:2000), TUBB4 (1:500). GAPDH primary antibodies were used for loading control (1:4000). The membranes were washed and incubated with horseradish peroxidase-conjugated goat anti-rabbit secondary antibodies (1:4000; Bio-Rad) at room temperature (RT) for 1 h. The signals were detected using enhanced chemiluminescence (Bio-Rad). ImageJ software (NIH) was used to analyze band intensities.

### Mitochondrial functional assays

Mitochondrial function was assessed by measuring the oxygen consumption rate (OCR) using a Seahorse XF-24 Flux Analyzer (Agilent Technologies, #103015-100). Briefly, the Stress test kit uses different electron transport chain inhibitors oligomycin, carbonyl cyanide-4-(trifluoromethoxy)phenylhydrazone (FCCP), and rotenone/antimycin A. Oligomycin inhibits ATP synthesis, preventing the passage of protons through the complexes. FCCP uncouples oxidative phosphorylation, while rotenone/antimycin inhibits mitochondrial complexes I and III, preventing respiration. PDCl-110 cells were treated with VERU-III or SB-216 for 24–72 hours and were seeded at 4 × 10^4^ cells/well in Seahorse XF24 Cell Culture Microplates. After adhering overnight, the cells were incubated with unbuffered DMEM (pH 7.4) supplemented with 2 mM L-glutamine, 1 mM pyruvate, and 10 mM glucose (Agilent Seahorse XF DMEM, #103575-100) at 37°C for 1 hour. OCR was measured under basal conditions and after sequential injections of oligomycin (1 μM), FCCP (1 μM), and rotenone/antimycin A (0.5 μM each). OCR values were normalized to total protein content, determined using a Bradford Protein Assay (Bio-Rad, #5000006).

### Statistical analysis

Data was analyzed using GraphPad Prism software. An unpaired parametric Student’s *t*-test was used to calculate the statistical significance between the two groups. One-way analysis of variance (ANOVA) was used to compare multiple groups. The statistical significance of the *in vitro* proliferation studies was determined using a two-way ANOVA. The results are presented as mean ± standard error of the mean (SEM), and the error bars represent SEM. Differences were considered statistically significant at *P*-values less than 0.05, with asterisks indicating ^*^p < 0.05, ^**^p < 0.01, ^***^p < 0.001, and ^****^p < 0.0001. Generally, we performed experiments three independent times and are presenting representative data.

## CONCLUSIONS

In the present study we demonstrate the effect of a novel strategy with dual-targeting inhibitor on PDAC cell growth. SB-216 significantly inhibits cell growth, reduces expression of oncogenic microtubule subtypes βIII and βIVb, as well as oncogenic mitochondrial function with inhibition of BRD4 in PDAC. SB-216 may play an important role in developing therapeutic agents that target mitochondrial function. SB-216 effectively inhibits PDAC cell growth through multiple therapeutic mechanisms, namely inhibiting oncogenic microtubules and mitochondrial function with a cancer-specific read out- PDAC cell growth. Future research on SB-216 will focus on the mechanistic relationship with mitochondrial bioenergetics and microtubule inhibition.
